# Investigating History of Suicidal Ideation Among Patients Attending Early Intervention for Psychosis Services: A Retrospective Analysis Using Clinical Records

**DOI:** 10.1192/bjo.2024.82

**Published:** 2024-08-01

**Authors:** David Mongan, Diego Quattrone, Ian Kelleher, Mary Cannon, David Cotter

**Affiliations:** 1 Queen's University Belfast, Belfast, United Kingdom; 2 Royal College of Surgeons in Ireland, Dublin, Ireland; 3 King's College London, London, United Kingdom; 4 University of Edinburgh, Edinburgh, United Kingdom

## Abstract

**Aims:**

Previous population-based studies have identified suicidal ideation (SI) as a potential risk marker for psychosis. We aimed to investigate the prevalence of previous SI in a large sample of patients with first episode of psychosis accepted to early intervention services (EIS) in South London and Maudsley (SLaM) NHS Foundation Trust using clinical records. We further aimed to investigate differences in patients with and without recorded SI according to age at diagnosis, gender, ethnicity and neighbourhood deprivation.

**Methods:**

We designed a retrospective cohort using the Clinical Record Interactive System. Included were patients who were accepted by SLaM EIS from 2015–2018 and received a psychotic disorder diagnosis (n = 1658). We used a natural language processing algorithm that searches deidentified textual clinical records, returning a binary variable indicating presence or absence of SI recorded at any time prior to acceptance to EIS. The algorithm has high precision (97%) and inter-rater reliability (Cohen's k 92%). The t-test was used to compare mean age at first diagnosis in patients with and without recorded SI, while chi-squared tests evaluated differences according to gender, ethnicity and tertiles of index of multiple deprivation (based on 2015 postcode). The significance threshold was p = 0.05.

**Results:**

The cohort included 1658 patients, of whom 656 (39.6%) were female. The natural language processing algorithm identified 600 patients (36.2%) who had SI recorded in their clinical records at any time prior to acceptance by EIS. On average, patients with recorded SI were younger at first diagnosis of psychotic disorder (mean 27.7 years, standard deviation 10.5) compared with patients without recorded SI (mean 30.1 years, standard deviation 11.2; p < 0.001). There was little evidence for differences on gender (p = 0.950), ethnicity (p = 0.059) or deprivation index (p = 0.597).

**Conclusion:**

Approximately 1 in 3 patients attending SLaM EIS had evidence of SI recorded prior to acceptance by EIS. Consistent with previous studies, the current findings emphasise the high prevalence of SI in this clinical population. Compared with those without SI, patients with recorded SI were on average 2–3 years younger at diagnosis. This may reflect general population age differences in prevalence of suicidal ideation; increased severity of illness with earlier age of onset; or patterns of contact with services which facilitated earlier diagnosis. There was little evidence that patients with and without recorded SI differed significantly on gender, ethnicity or neighbourhood deprivation. Prospective studies would be helpful to assess whether SI is a risk marker for first episode of psychosis.

